# Human reliability of the intelligent construction site tower crane driver interface based on DEMATEL-ISM-BN

**DOI:** 10.1371/journal.pone.0303996

**Published:** 2024-10-17

**Authors:** Niu Lixia, Wen Si

**Affiliations:** School of Business Administration, Liaoning Technical University, Xingcheng City, Liaoning Province, China; Gonbad Kavous University, ISLAMIC REPUBLIC OF IRAN

## Abstract

With the arrival of Industry 4.0, intelligent construction sites have seen significant development in China. However, accidents involving digitized tower cranes equipped with smart systems continue to occur frequently. Among the main causes of these accidents is human unsafe behavior. To assess the human factors reliability of intelligent construction site tower cranes, it is necessary to shift the safety focus to the human-machine interface and identify patterns of human error behaviors among tower crane drivers through text mining techniques (TF-IDF-TruncatedSVD-ComplementNB). Based on the SHEL model, the behavioral factors influencing human factors reliability in the human-machine interface are categorized and a Performance Shaping Factors (PSF) system is constructed. Building on the foundation of constructing an indicator system for human factors error influence in the driver interface of intelligent construction site tower cranes, the Decision-Making Trial and Evaluation Laboratory (DEMATEL) method is combined with the Interpretive Structural Modeling (ISM) to analyze the importance of various factors in causing human errors and to analyze the logical structure among these factors. Simultaneously, a Bayesian network is constructed using a multi-level hierarchical structural model, thus establishing a new evaluation method for the human-machine interface. The effectiveness of the proposed method is validated through Bayesian network causal inference based on real case studies. The results demonstrate that the evaluation process of this method aligns with the operational scenarios of tower crane drivers in intelligent construction sites. It not only allows for quantifying the likelihood of human errors but also enables the development of targeted measures for controlling unsafe behaviors among tower crane drivers in intelligent construction sites.

## Introduction

Smart construction sites utilize modern information technology to achieve high levels of connectivity between people, objects, and machines, incorporating safety concepts into the production process and improving production efficiency and safety management goals [1]. Currently, many tower cranes in construction sites are equipped with hook visualization devices, effectively reducing the likelihood of hooking and collision accidents [2]. Compared to traditional construction sites, the widespread adoption of hook visualization in tower cranes on smart construction sites requires drivers to have a higher situational awareness [3]. According to statistics, there were a total of 605 tower crane accidents in China from 2016 to 2020, averaging about 121 accidents per year [4]. Distraction has been identified as a common cause of crane accidents, accounting for 19% of tower crane accidents [5], and hook visualization may increase the driver’s potential for distraction [6]. As the information and automation level of tower cranes improve, the unsafe conditions of objects have decreased, but human unsafe behaviors have become the main cause of safety accidents [7]. Unsafe behaviors of workers not only directly cause safety accidents but can also indirectly lead to accidents by altering the state of objects [8].The addition of interface management tasks imposes additional cognitive and operational loads on the driver, increasing the likelihood of human errors, such as mode confusion and loss of situational awareness [9]. Therefore, the reliability and safety of the human-machine interaction system equipped with hook visualization tower cranes increasingly depend on human factors [10], and timely analysis of the causes of accidents involving smart tower cranes has been an important concern in the construction industry.

Currently, academic research on human errors in traditional tower cranes has mainly focused on exploring the factors influencing safety accidents. However, few researchers have investigated the interactions among safety influencing factors and human reliability in tower cranes equipped with hook visualization devices on smart construction sites. Some researchers have used Rasmussen’s risk management theory to identify 56 influencing factors related to tower crane safety and have constructed a tower crane safety universal model using the AcciMap technique to demonstrate the causal paths between system levels and influencing factors [11]. In addition, other scholars have used a framework approach to systematically analyze the causes and influencing factors of tower cranes accidents in the Australian construction industry. They have identified 77 contributing factors and found that these factors operate on multiple levels of the work system associated with tower crane use [12]. Another study developed the Framework for Fuzzy Integrated Risk Analysis (ERAFF), which aims to provide an overview of critical causal factors and risks and control measures in the overall framework to improve the safety of tower crane operations [13]. Furthermore, some researchers have regarded all the causes of tower crane accidents as a system and used network analysis methods to divide them into six subsystems and 34 factors. They determined seven key factors and three key paths of tower crane accident causes by calculating statistical indicators such as degree, strength, and shortest path of the network model [14]. With the advent of Industry 4.0, many traditional tower cranes in China have gradually installed hook visualization monitoring systems, improving safety factors and optimizing performance. The reliability and safety of human-machine interaction systems increasingly depend on human factors [10] and human reliability assessment is a technique for assessing the impact of human errors on systems. It can identify potential human errors and their causes to reduce the probability of human error occurrences.

Multi-Criteria Decision Making (MCDM) techniques are essential for addressing complex decision problems with conflicting criteria [15]. While traditional methods like AHP and TOPSIS are well-established, recent advancements have introduced novel approaches to enhance decision-making [16]. COPRAS evaluates alternatives relative to ideal solutions [17], while WASPAS provides a comprehensive assessment through weighted summation and product aggregation [18]. SECA offers a holistic perspective by simultaneously evaluating criteria and alternatives [19]. Additionally, CODAS ranks alternatives based on their distance from the ideal solution [20], SWARA II determines criteria weights through pairwise comparisons, and MEREC evaluates criterion importance by assessing the impact of removing individual criteria [21]. EDAS ranks alternatives based on their distance from the average solution, considering both ideal and anti-ideal solutions [22]. These methods offer diverse approaches to address various decision-making contexts. The Decision Making Trial and Evaluation Laboratory (DEMATEL) helps to reveal causal relationships, providing a deeper understanding, while Interpretive Structural Modeling (ISM) captures interrelationships among factors, emphasizing their importance within the entire system architecture [23]. Considering that People, Systems, and Task Features (PSFs) exhibit causal relationships and interdependencies, the DEMATEL-ISM method is chosen for decision analysis [24]. Due to limited empirical data on accidents involving intelligent tower cranes, this study relies more on expert assessments of the reliability of lifting operations, focusing primarily on the interaction between intelligent tower crane operators and machines.By text mining 229 accident reports involving traditional tower cranes and intelligent tower cranes (2018–2023), the SHEL model is used to categorize human-machine factors within the crane operator’s cab into 28 aspects, including physical fitness, fatigue level, level of attention, emotional state, knowledge skills, and operational abilities. A factor questionnaire survey is conducted, with factor S10 excluded. Furthermore, the DEMATEL-ISM method is applied for the first time to assess human errors in intelligent tower cranes at construction sites. Through an expert survey form, the DEMATEL method is used to calculate the comprehensive influence matrix of indicators, and an ISM model is constructed to build a topological network. Based on this, a Bayesian network is established, and the Softmax normalized Bayesian network is utilized to convert word frequencies obtained from text mining into probabilities. An example is provided to validate the reliability analysis method for human errors in intelligent construction site tower cranes.Although limitations exist regarding accident data involving intelligent tower cranes at construction sites, expert assessments play a crucial role in the study. Further research and practice will contribute to a more comprehensive and accurate assessment of the reliability of human factors in intelligent tower cranes at construction sites, providing targeted measures and solutions to reduce human errors.

## Research methodology

### Text mining for tower crane accidents

Due to the limited and relatively novel research on smart construction site tower cranes, the selection of performance influencing factors cannot solely rely on previous tower crane accidents. Therefore, a combination of text mining methods and the SHEL model can be utilized to determine evaluation indicators. By employing text mining techniques to understand the accident features of tower cranes in smart construction sites, it can enhance the hierarchy of indicators and reduce overlap, thereby facilitating subsequent accurate calculations. The development of smart construction sites in China started relatively late, with large-scale construction beginning around 2016. However, the practical application and promotion of smart construction sites have gradually matured in recent years. Currently, the development of smart construction sites in China is rapidly progressing and has become a new trend in construction site management. According to data released by the Ministry of Industry and Information Technology, as of the end of 2020, there were 1023 smart construction sites in China, with Shanghai, Guangdong, Tianjin, Zhejiang, and Jiangsu having the highest number of smart construction sites. Currently, most tower cranes in smart construction sites are equipped with hook visualization systems, resulting in lower accident rates and fewer accident reports. Therefore, a total of 229 tower crane accident reports (including both traditional and smart tower cranes) from 2018 to 2023 were collected from the website of the State Administration of Work Safety and the Crane Engineer website. These reports were used as the data source to ensure the authority, accuracy, and timeliness of the data. However, tower crane accident reports often lack standardization and consistency, leading to redundant information. Given the semantic complexity and sparsity of long texts in text mining, text preprocessing is initially performed to address these characteristics. Irrelevant information, such as details about the accident unit, improvement suggestions, and the accident investigation process, has been removed to focus specifically on unsafe behaviors and their causes. Only the accident process, accident causes, and accident liability attribution were retained and integrated for subsequent text mining.

First, the tower crane accident text corpus was preprocessed using Python by removing non-Chinese characters, tokenizing, and eliminating stop words. This step involved constructing a tower crane safety feature dictionary. Next, the inverse document frequency (IDF) value of each word was calculated to build a custom IDF dictionary, enhancing the accuracy of keyword extraction. The TF-IDF algorithm was then applied to extract keywords from all the tower crane safety texts collected [25]. Using the constructed tower crane safety domain feature dictionary, feature matching was performed on the extracted keywords, obtaining the feature attributes of each tower crane safety accident text. Since the text contains nearly 600,000 words and yields a large number of feature words through TF-IDF, TruncatedSVD was selected to reduce dimensionality, resulting in 127 representative feature words [26]. When faced with significant disparities between indicators derived from text mining and expert interviews, two potential explanations arise [27]. Firstly, differences may stem from limitations inherent in text mining algorithms, such as variations in tokenization processes and difficulties in handling domain-specific terms and data sparsity. Secondly, limitations in experts’ understanding of the problem context could contribute to disparities. To address these issues, a comprehensive analysis of disparate indicators is necessary. This involves:

1. Reviewing literature and incident reports related to specific indicators to determine their significance.
2. Seeking input from third-party experts to validate indicators with significant disparities.
3. Verifying newly proposed indicators by experts through alignment with literature and incident reports.
4. Requesting rationales from experts for dissenting opinions on indicators and investigating supporting evidence in literature or incident reports.
5. Collaborating with experts to decide on the inclusion or exclusion of disputed indicators based on evidence.
6. Removing indicators lacking supporting literature or incident reports [28].

In cases of substantial disparity, redesigning the expert interview methodology may be necessary to ensure clearer and more precise questioning, minimizing subjective influences. Finally, the ComplementNB class was used to train the Naive Bayes classifier with the obtained features and target variables. The model parameters of the classifier were fitted, and predictions were made, producing an accuracy output of 1. This preliminary result indicates that the model possesses accuracy and generalization capabilities on the training set [29]. Due to the abundance of feature words, 97 representative features were selected through sorting after manually excluding irrelevant items such as "construction" and "safety management." Expert opinions from the construction industry were sought, suggestions were considered and discussed, and relevant literature was reviewed [30]. The 97 feature values were chosen based on their representativeness and weight, and the encoding results are summarized in Table 1.

**Table 1:** Dimension reduction results of the feature items of the intelligent site tower crane accident investigation report.

Impact Factors	Frequency	Impact Factors	Frequency	Impact Factors	Frequency
Feel unwell	0.067	Teamwork	0.067	Cockpit temperature	0.0034
Physical state	0.067	Teamwork	0.067	Cockpit humidity	0.0034
Illness	0.034	Operating specification	0.0034	Illumination	0.0034
Health	0.034	Operating system	0.0034	Hue	0.0034
Fatigued	0.034	Rules and regulations	0.0034	Noise	0.0034
Dispersion of attention	0.034	Reward and punishment system	0.0034	Vibration	0.0034
distract	0.034	Management system	0.0034	Crossing condition	0.0034
Inattention	0.034	Job training	0.0034	Construction site	0.0034
Emotional stability	0.034	Safety education	0.0034	Site obstacle	0.0034
Testiness	0.034	Job management	0.0034	Weather	0.0034
Safety awareness	0.034	Operating procedure	0.0034	Digital interface display	0.0034
Professional skill	0.034	Technical specification	0.0034	Digital interface information delivery	0.0034
Operational skill	0.034	Regulation	0.0034	Information transmission	0.0034
Defense	0.034	Long working hours	0.0034	Safety sign	0.0034
Safety belt	0.034	The work schedule is not reasonable	0.0034	Display and control page layout	0.0034
Safety Helmet Hat	0.034	Cable worker communication	0.0034	Display and control operation mode	0.0034
Safety measure	0.034	Signalman	0.0034	Display and control device density	0.0034
Protective device	0.034	Untimely signal	0.0034	Drive-by-wire reliability	0.0034
Working hours	0.135	Improper command	0.0034	Space comfort	0.0034
Staffing	0.034	Emergency drill	0.0034	Cockpit seat comfort	0.0034
Distribution of responsibilities	0.034	Emergency plan	0.0034	Communication equipment	0.0034
Time pressure	0.034	Preventive measures	0.0034	System intelligence	0.0034
Time shortage	0.034	Working atmosphere	0.0034	System reliability	0.0068

### Identification of behavior formation factors

Based on the SHEL model frequently used in the field of aviation safety [31], the elements covering the human-machine interface of the intelligent construction site tower crane are divided into four aspects: System Personnel (L), System Software Operating Specifications (S), System Hardware (H), and System Environment (E). The focus of the assessment is to determine the relationships between L-L, L-S, L-H, and L-E. Through text mining, we obtained indicators and conducted literature reviews and expert interviews, resulting in a total of 28 PSFs as shown in Table 2. The SHEL model is commonly employed in the field of aviation safety to assess and study the impact of interactions between personnel, software, hardware, and environment on system safety. In the context of studying the human-machine interface of the intelligent construction site tower crane, this model is applied to determine the relationships between system personnel, software operating specifications, system hardware, and system environment, in order to assess and improve these elements. Table 2 presents the 28 PSFs indicators obtained through text mining, literature reviews, and expert interviews. These indicators provide valuable information about various aspects of the human-machine interface of the intelligent construction site tower crane and can be utilized for further research and analysis.

**Table 2:** Summarizes the PSFs of the human-machine interface of the smart construction tower crane.

Subsystem	Human error factor	Index	Instructions	Literature reference
**L-L**	Physical fitness	Physical discomfort, physical condition, disease	Whether the personal physical quality is good, such as eyesight, physical coordination and other good degree	[32]
	Fatigue degree	fatigued	An individual’s subjective perception of physical fatigue	[33]
	Concentration level	Distraction, inattention, distraction	Personal focus on work	[34]
	Emotional state	Emotionally stable and irritable	The expression of personal psychological emotions, such as calm, excitement, etc	[35]
	Knowledge skills and business ability	Safety awareness, professional skills, operational skills	The amount of relevant driving knowledge, the level of mastery of driving skills and the awareness of the situation	[36]
	Wear personal protective equipment	Protective equipment, safety belts, safety hats, safety measures, protective devices	Whether individuals can properly use protective equipment and whether enterprises can provide protective equipment	[37]
	Clear division of labor and responsibilities	Division of labor, staffing, responsibility distribution	Team members for their own role positioning and job responsibilities clear degree	[38]
	Time pressure	Time pressure, time shortage	Whether the crane driver can complete the task within a limited time	[39]
	Degree of teamwork	Teamwork, teamwork	The quality of information exchange and operation cooperation between team members	[40]
**L-S**	System completeness of operating standards	Operation standard, operation system	Organization and management, operating standards and systems are scientific and perfect	[41]
	The rationality of reward and punishment system	Rules and regulations, reward and punishment system, management system	Whether the reward and punishment system can effectively mobilize the enthusiasm of drivers to operate the software	[42]
	Completeness of tower crane operating procedures	Complete operating procedures, technical procedures and procedures	Whether the procedures and norms for executing operational tasks are complete	[43]
	Working time rationality	Long working hours and unreasonable working schedule	The working and rest time arrangement of team members and the duration of continuous work are reasonable	[33].
	Communicate properly with the operator	Cable worker communication, signal worker, signal is not timely, improper command	Able to communicate clearly and reasonably with the operator	[44]
	Emergency drills and plans	Emergency drills, emergency plans, preventive measures	The frequency and quality of emergency drills and the adequacy of safety plans for emergencies	[44]
	Working atmosphere	Working atmosphere	The working atmosphere of the team is harmonious	[45]
**L-E**	Microclimate	Cab temperature, cab humidity	Whether the micro climate composed of air pressure, temperature, humidity and ventilation is conducive to the physiological comfort of personnel	[46]
	Lighting, color	Lighting, color	Whether the lighting and color are conducive to the visual recognition function of personnel, visual information exchange, etc	[47]
	Noise and vibration	Noise and vibration	Whether noise and vibration are beneficial to people’s auditory sensitivity, manipulation accuracy and emotional state	[40]
	Crossing conditions, site obstacles	Crossing condition	Crossing conditions and site obstacles refer to the situations in which different traffic flows or different vehicles cross and meet each other in the road surface system	[32]
	Abnormal climate change	Weather anomaly	Weather The temperature changes greatly and the weather is abnormal.	[48]
**L-H**	The clarity, legibility and reliability of digital interface information transmission	The device interface information is poorly designed, the device is over-used/fatigued, and the device panel alarm is not clear	The prominence of important information display in the digital interface, the speed of obtaining information, text, icon symbols	[49]
	Display and control device layout	Display and control page layout, display and control operation mode, display and control device density, display and control device reliability	The visibility of the display device, the location accessibility of the control device, the corresponding relationship between the display and control combination layout, and whether the function division conforms to the experience and expectations of the personnel	[50]
	mandatory sign	Safety sign	The accuracy and differentiation of relevant indication symbols and signs	[51]
	Overall spatial layout	Space comfort, vision, operating lever	Structural dimensions of work areas, channels, and activity Spaces	[48]
	Cab seat	Cab seat	The degree to which the structure of the seat matches the operation of the sitting position and the comfort of the human spine	[35]
	Communication equipment	Communication equipment	The good working condition of communication equipment, the stability and clarity of communication signals	[36]
	System automation level	System intelligence, system reliability	Automation system reliability, equipment complexity	[48]

1. L-L Relationship: Study on the information exchange and collaborative capacity between the crane operator and the team members.
2. L-H Relationship: Research on the interaction between the crane operator and the hardware operating equipment.
3. L-S Relationship: Investigation of the human-machine relationship between the crane operator and the team management, technical training, and operational standards.
4. L-E Relationship: Examination of the relationship between the crane operator and the operating environment of the driver’s cabin.

### Survey on behavior formation factors

To establish a human reliability Performance Shaping Factors (PSFs) system for the human-machine interface of smart construction tower cranes, a survey questionnaire was used to investigate the 28 identified behavior formation factors. The questionnaire was distributed to male participants, with experience operating tower cranes in smart construction sites where visualized hooks were used, mainly in Shanghai, Shenzhen, and Hubei. The participants had an average age of around 40 years. Through the analysis of questionnaire data, the survey provided strong support for constructing the PSFs system. The questionnaire included basic information and an investigation of the PSFs that influence human reliability, using a Likert 5-point scale where 1 represented "minimal impact" and 5 represented "significant impact". A pilot survey was conducted before distributing the formal questionnaire to ensure its validity. A total of 137 questionnaires were collected, with 132 of them considered as valid. A reliability test was conducted to ensure the data’s validity.

The study is exempt from the need for approval as it is a low-risk survey study.This study was conducted in accordance with the guidelines outlined in the Declaration of Helsinki. All participants provided informed consent prior to their participation in the study. For participants under the age of 18, parental consent was obtained. The consent process included a clear explanation of the study’s purpose, procedures, potential risks, and benefits. Participants were informed of their right to withdraw from the study at any time without consequences.To ensure the anonymity of the subjects, all personal identifying information was removed or disguised during data analysis and reporting. Each participant was assigned a unique identifier to maintain confidentiality. Only aggregated data without individual identifiers are presented in this manuscript to protect the privacy of the participants.

The questionnaire was assessed for its reliability and validity:

① Using SPSS software, the overall reliability of the questionnaire was found to be 0.982, with reliability coefficients of 0.941 (L-L), 0.895 (L-H), 0.940 (L-S), and 0.937 (L-E) for the four dimensions, indicating good consistency across all dimensions.

② Content validity, correlation calibration validity, and structural validity of the questionnaire were examined. The KMO (Kaiser-Meyer-Olkin) test value for the questionnaire data was 0.974, and Bartlett’s spherical test’s approximate chi-square value was 3779.906. The communalities for all research items were above 0.4, indicating that the information from the research items could be effectively extracted. Additionally, the KMO value was 0.963, which is higher than 0.6, indicating effective information extraction from the data. The variance interpretation rates of the four factors were 25.172%, 21.044%, 18.427%, and 11.352%, respectively, with cumulative variance interpretation rates of 68.388%, 70.481%, 72.498%, and 74.265% after rotation, suggesting that the information from the research items could be effectively extracted. Items with scores lower than 0.5 were filtered out through principal component analysis. In this study, S10 (operational standard completeness) did not meet the research conditions and was therefore excluded.

### Behavior formation factors system

**Fig 1:**
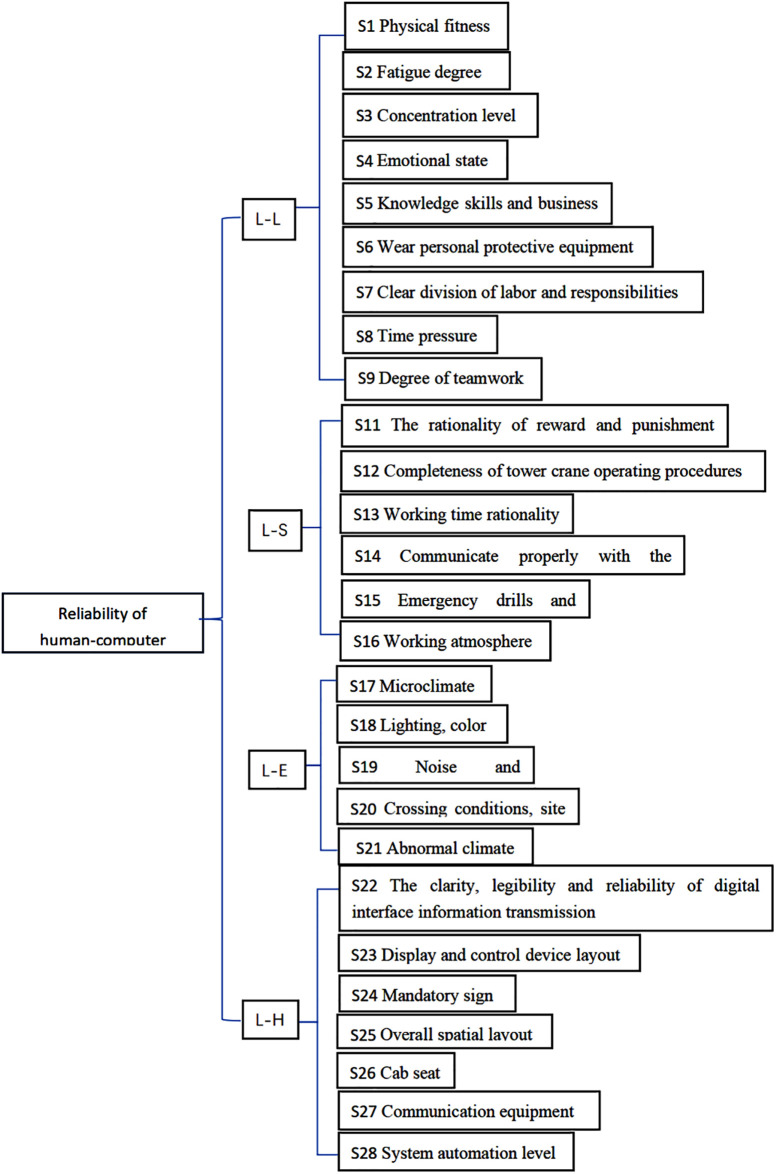
Behavior formation factor (PSF) system.

Through the analysis of the questionnaire data, a PSFs system consisting of four dimensions was finalized. This set of indicators can reflect the impact of the human-machine interface in the tower crane driver’s cabin on the operator’s behavior, as shown in Fig 1.

### DEMATEL-ISM-BN model

The DEMATEL-ISM-BN model combines expert knowledge and utilizes graph theory and matrix theory to describe the strength of interrelationships between various influencing factors. It calculates indicators such as influence degree, being influenced degree, cause degree, and centrality to identify key elements in complex systems. ISM reflects the intrinsic relationships between influencing factors in a complex system through reachable matrices and constructs a multi-level hierarchical structure model. It visually represents the hierarchical relationships between influencing factors through a topological graph. BN, based on Bayesian theorem, establishes a probabilistic network that focuses on probabilistic inference and enables prediction of a certain observation point through probabilistic statistical methods [52]. By combining the comprehensive influence matrix from DEMATEL with the identity matrix, the overall influence matrix is obtained. Through calculations, it can be transformed into the reachable matrix required by ISM. Compared to using ISM alone, this approach not only reveals the relationships between influencing factors but also reflects the strength of interactions between them and the extent of their impact on accidents [52]. Based on the ISM model, a multi-level hierarchical structure model is constructed and mapped into the BN model. By integrating practical cases, key factors can be extracted through probability distribution and posterior probability calculations.

The specific steps are as follows:

1. Determine the set of influencing factors. Through literature review and analysis of actual accident cases, similar or duplicate factors are merged to ultimately determine the set of influencing factors, S = {s1, s2, …, sn}.
2. Calculate the initial direct influence matrix, D. Based on expert knowledge and experience, the interrelationships between factors are obtained, resulting in the influence relationship matrix D = [dij]n × n. The matrix coefficient dij represents the direct influence of factor ai on factor aj.
$${\text{d}}_{\text{ij}}=\left\{\begin{array}{l} 0F\text{actor}\;\text{i}\;\text{has}\;\text{no}\;\text{effect}\;\text{on}\;\text{j}\\ 1F\text{actor}\;\text{i}\;\text{has}\;\text{weak}\;\text{effect}\;\text{on}\;\text{j}\\ 2F\text{actor}\;\text{i}\;\text{has}\;\text{strong}\;\text{effect}\;\text{on}\;\text{j}\\ 3F\text{actor}\;\text{i}\;\text{has}\;\text{the}\;\text{strongest}\;\text{effect}\;\text{on}\;\text{j} \end{array}\right.$$ (1)
  When i = j, dij = 0.
3. Normalize the direct influence matrix. Normalization is performed on the direct influence matrix D to obtain the normalized direct influence matrix C, as shown in Eq (2).
$$C={\left[c_{ij}\right]}_{n\times n}=\frac{1}{\underset{1\leq p\leq n}{\text{max}}\sum_{q=1}^{n}D_{ij}}D$$ (2)
4. Solve the comprehensive influence matrix T to identify the most critical factors, as shown in Eq (3). Here, I represents the identity matrix, indicating the influence of factors on themselves.
$$T=C+C^{2}+C^{3}+C^{4}+\cdots C^{n}=C(I-C^{n-1})/I-C={\left[t_{ij}\right]}_{n\times n}$$ (3)
5. Based on the comprehensive influence matrix T, calculate the impact degree fi, being influenced degree ei, centrality zi, and cause degree yi of each influencing factor, as shown in Eqs (4)–(7).
$$f_{i}=\sum_{j=1}^{n}t_{ij}(i=1,2,\ldots ,n)$$ (4)
$$e_{i}=\sum_{j=1}^{n}t_{ij}(i=1,2,\ldots ,n)$$ (5)
$$z_{i}=f_{i}+e_{i}(i=1,2,\ldots ,n)$$ (6)
$$y_{i}=f_{i}-e_{i}(i=1,2,\ldots ,n)$$ (7)
6. Calculate the reachable matrix. By calculating I + T, the overall influence matrix is obtained. The reachable matrix in the ISM model is determined, where kij is calculated as shown in Eq (8). Here, the threshold λ = α + β (α and β are the mean and standard deviation of elements in matrix T).
$${\text{k}}_{\text{ij}}=\left\{\begin{array}{l} 1h_{ij}>\lambda \\ 0h_{ij}\leq \lambda \end{array}\right.$$ (8)
7. Hierarchical structure analysis. Based on the reachable matrix K, the reachable set R(Si) and the antecedent set A(Si) can be obtained. R(Si) represents the set of columns in the reachable matrix K where the i-th row factor contains the element 1, while A(Si) represents the set of rows in the reachable matrix K where the i-th column factor contains the element 1. The hierarchical division of the system is performed according to Eq (9).
$$R(S_{i})=R(S_{i})\cap A(S_{i}),i=1,2,\ldots ,n$$ (9)

If the discriminant formula holds true, it indicates that the corresponding factor "a" is a bottom-level factor, and the row and column containing this factor are deleted from the reachable matrix K. The remaining factors repeat step 7) until all factors are deleted.The factors are shown in Table 3.

**Table 3:** Reachable set and antecedent set of a certain factors.

Factors	R (Si)	A (Si)	R (Si) ∩ A (Si)	Distinguish
**a**	{a}	{a,b,c}	{a}	R (Si) = R (Si) ∩ A (Si)
**b**	{a,b,c}	{b,c}	{b,c}	R (Si) ≠R (Si) ∩ A (Si)
**c**	{a,c}	{b,c}	{c}	R (Si) ≠R (Si) ∩ A (Si)

### Analysis of human error factors in driver interface using DEMATEL

Considering the initial 27 indicators, conducting DEMATEL analysis would require pairwise comparisons in two directions, resulting in a need for 676 columns of indicator data. This would not only pose difficulties for the experts but also lead to distorted responses in the survey questionnaire, potentially causing significant issues in subsequent calculations. Therefore, based on expert experience and literature research, the 27 indicators were consolidated into 7 main factors to simplify the analysis.

The L-L relationship can be divided into personal state and personal abilities. Personal state encompasses physical fitness, level of fatigue, concentration level, and emotional state. Personal abilities include knowledge, skills, and proficiency, personal protective equipment usage, clarity of personnel roles and responsibilities, and time pressure.

The L-S relationship can be divided into organizational management and safety culture. Organizational management includes team collaboration, completeness of operating procedures, rationality of reward and punishment systems, and completeness of tower crane operating regulations. Safety culture covers aspects such as reasonable working hours, effective communication with signallers, emergency drills and plans, and work atmosphere.

The L-E relationship and L-H relationship can be respectively categorized as the physical environment and technological environment. The results are shown in Table 4.

**Table 4:** System of human error factors in driver interface.

Target layer	Criteria layer	Indicator layer
	L-L	T11 Personal status
		T12 Personal ability
**Human Error**	L-S	T21 Organization and Management
		T22 Safety culture
		T23 Operating procedures
	L-E	T3 Physical Environment
	L-H	T4 Technical environment

Invitation to 10 researchers in the field of construction engineering to participate in a questionnaire survey. The researchers are asked to provide bidirectional ratings for 7 indicators of influence based on their personal experience and professional knowledge. Based on the questionnaire results and expert opinions, the direct influence matrix D is calculated. The comprehensive influence matrix is then computed using formulas (1) and (2). Specific data can be found in. Table 5, modified comprehensive influence matrix in Table 6.

**Table 5:** Direct influence matrix.

	**T11**	**T12**	**T21**	**T22**	**T23**	**T3**	**T4**
**T11**	0	0	0	0	1	1	1
**T12**	0	1	0	0	0	0	0
**T21**	2	1	0	1	1	0	0
**T22**	3	2	2	0	1	0	0
**T23**	2	1	2	2	0	1	1
**T3**	2	1	2	2	1	0	0
**T4**	2	0	2	2	1	0	0

**Table 6:** Modified comprehensive influence matrix.

	**T11**	**T12**	**T21**	**T22**	**T23**	**T3**	**T4**
**T11**	0.185	0.093	0.140	0.127	0.195	0.153	0.153
**T12**	0.000	0.125	0.000	0.000	0.000	0.000	0.000
**T21**	0.390	0.223	0.119	0.199	0.204	0.066	0.066
**T22**	0.547	0.368	0.345	0.132	0.244	0.088	0.088
**T23**	0.591	0.333	0.447	0.406	0.205	0.200	0.200
**T3**	0.537	0.314	0.406	0.369	0.277	0.090	0.090
**T4**	0.537	0.189	0.406	0.369	0.277	0.090	0.090

**Fig 2:**
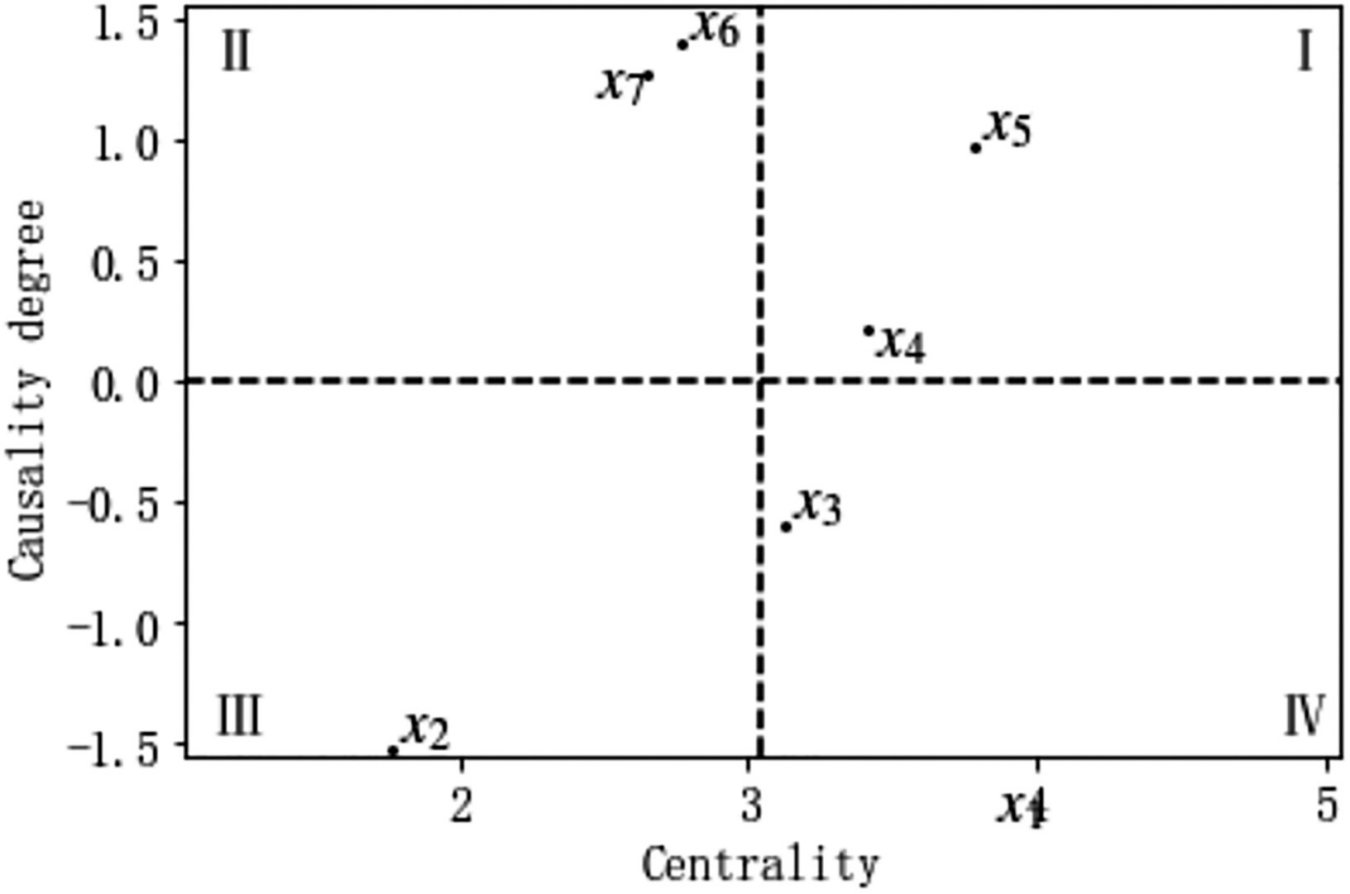
Distribution of the attribute "degree centrality" of the influencing factors for driver interface-related human errors. Note:x1 corresponds to T11,x2 corresponds to T12,x3 corresponds to T21,x4 corresponds to T22,x5 corresponds to T23,x6 corresponds to T3 and x7 corresponds to T4.

The Python program calculates the influence degree, affected degree, causality degree, and centrality of each factor, as shown in Table 7. A causality degree greater than 0 is considered a causal factor, while a causality degree less than 0 is considered a result factor. Causal factors have a significant influence on other factors, while result factors are more prone to being influenced by causal factors in the system. distribution of the attribute "degree centrality" of the influencing factors for driver interface-related human errors, as shown in Fig 2.

**Table 7:** DEMATEL analysis results for driver interface human error factors.

Influence factor	Influence degree	Affected degree	Causality degree	Factor attribute	Centrality	Centrality ranking
**T11**	1.047	2.788	-1.741	Outcome factor	3.835	7
**T12**	0.125	1.645	-1.520	Outcome factor	1.770	1
**T21**	1.268	1.862	-0.595	Outcome factor	3.130	4
**T22**	1.812	1.602	0.210	Cause factor	3.414	5
**T23**	2.380	1.403	0.977	Cause factor	3.783	6
**T3**	2.084	0.688	1.396	Cause factor	2.772	3
**T4**	1.959	0.688	1.271	Cause factor	2.647	2

For the human error factors in the intelligent construction site tower crane, the causal factors are ranked in descending order of causality degree as T3 physical environment, T4 technical environment, T23 operational procedures, and T22 safety culture. The result factors are ranked in descending order as T11 personal state, T12 personal competence, and T21 organizational management. Therefore, T3 physical environment and T4 technical environment are the main driving factors leading to the occurrence of other factors. The higher the centrality, the greater the importance. Hence, T11 personal state, T23 operational procedures, and T22 safety culture are the main factors contributing to human error.

**Fig 3:**
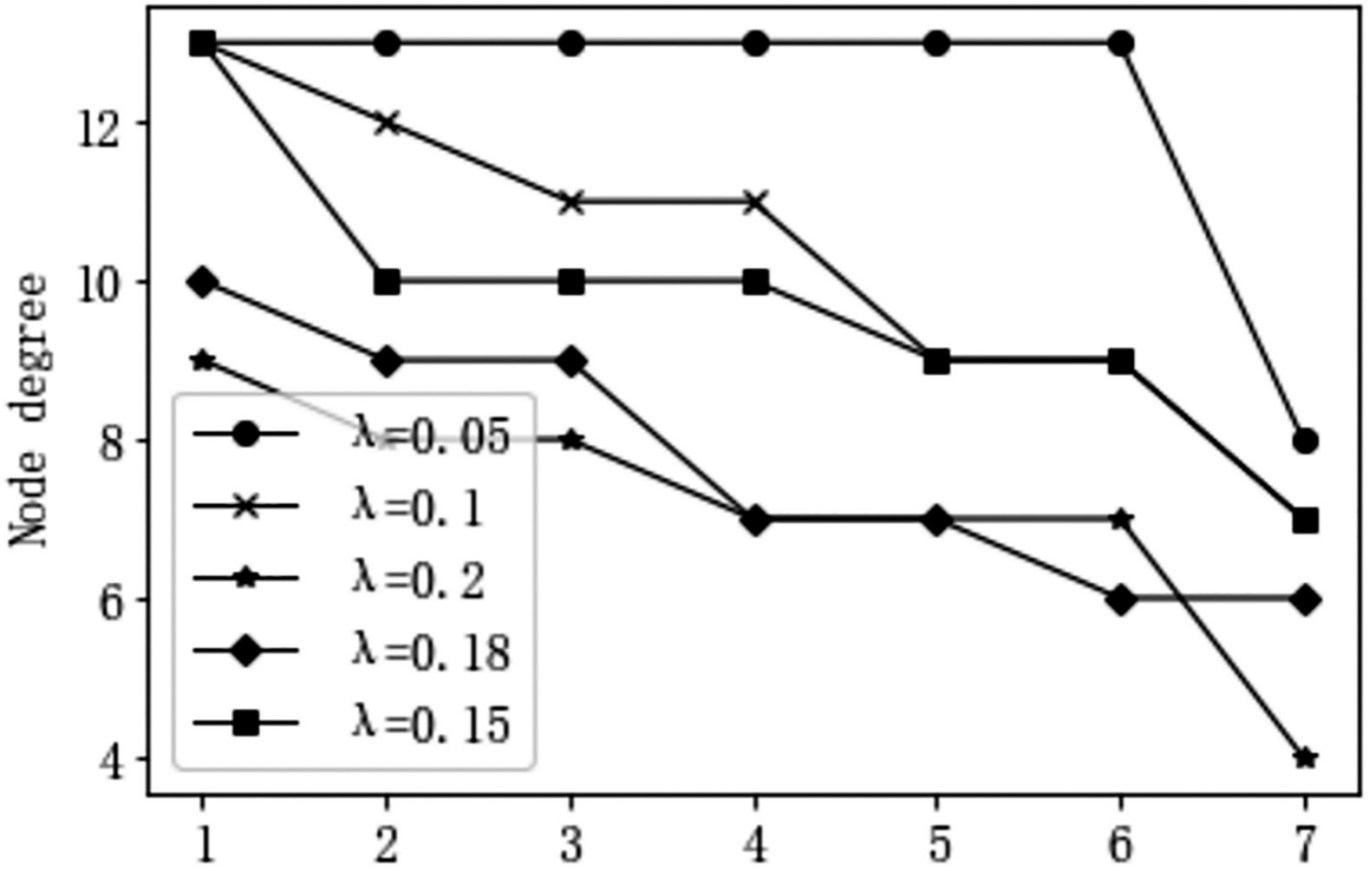
Node degree—Influencing factor diagram for driver interface-related human errors at different thresholds.

Based on the comprehensive influence matrix, the overall influence matrix is established by considering the self-influence factors (incorporating the identity matrix). In the process of transforming the overall influence matrix into a reachable matrix, it is necessary to set an appropriate threshold to eliminate relationships with low influence between factors, thereby ensuring moderate degrees of the factor nodes. Traditional methods mainly rely on empirical multiple value selections to determine the threshold and obtain satisfactory results. As shown in Fig 3, through multiple value analyses, it is observed that when the threshold λ is set to 0.15, the node degrees are relatively appropriate, which is conducive to the hierarchical division of factors. Therefore, in this study, a threshold of 0.15 is selected, and the reachable matrix of the forming factors can be obtained based on the results of the overall influence matrix and Eq (8), as shown in Table 8.

**Table 8:** Reachable matrix K of the influencing factors for driver interface-related human errors.

**Factors**	**T11**	**T12**	**T21**	**T22**	**T23**	**T3**	**T4**
**T11**	1	0	1	0	1	1	1
**T12**	0	1	0	0	0	0	0
**T21**	1	1	1	1	1	0	0
**T22**	1	1	1	1	1	0	0
**T23**	1	1	1	1	1	1	1
**T3**	1	1	1	1	1	1	0
**T4**	1	1	1	1	1	0	1

### Construction of multi-level hierarchical model for influencing factors of driver interface-related human errors

To systematically partition the reachability matrix, this study adopts spectral clustering. Spectral clustering is a clustering algorithm based on graph theory and linear algebra. It treats the dataset as a graph, where each data point represents a node, and edges are constructed based on the similarity (or distance) between the nodes. By decomposing and clustering the graph spectrum, the data points are partitioned into different levels. Building upon the classification of the spectral clustering algorithm, the hierarchical structure of factors influencing human-induced errors in the driver interface of intelligent operation for construction site tower cranes is further refined based on expert experience. It consists of three levels, with complex hierarchical relationships between each factor.

**Fig 4:**
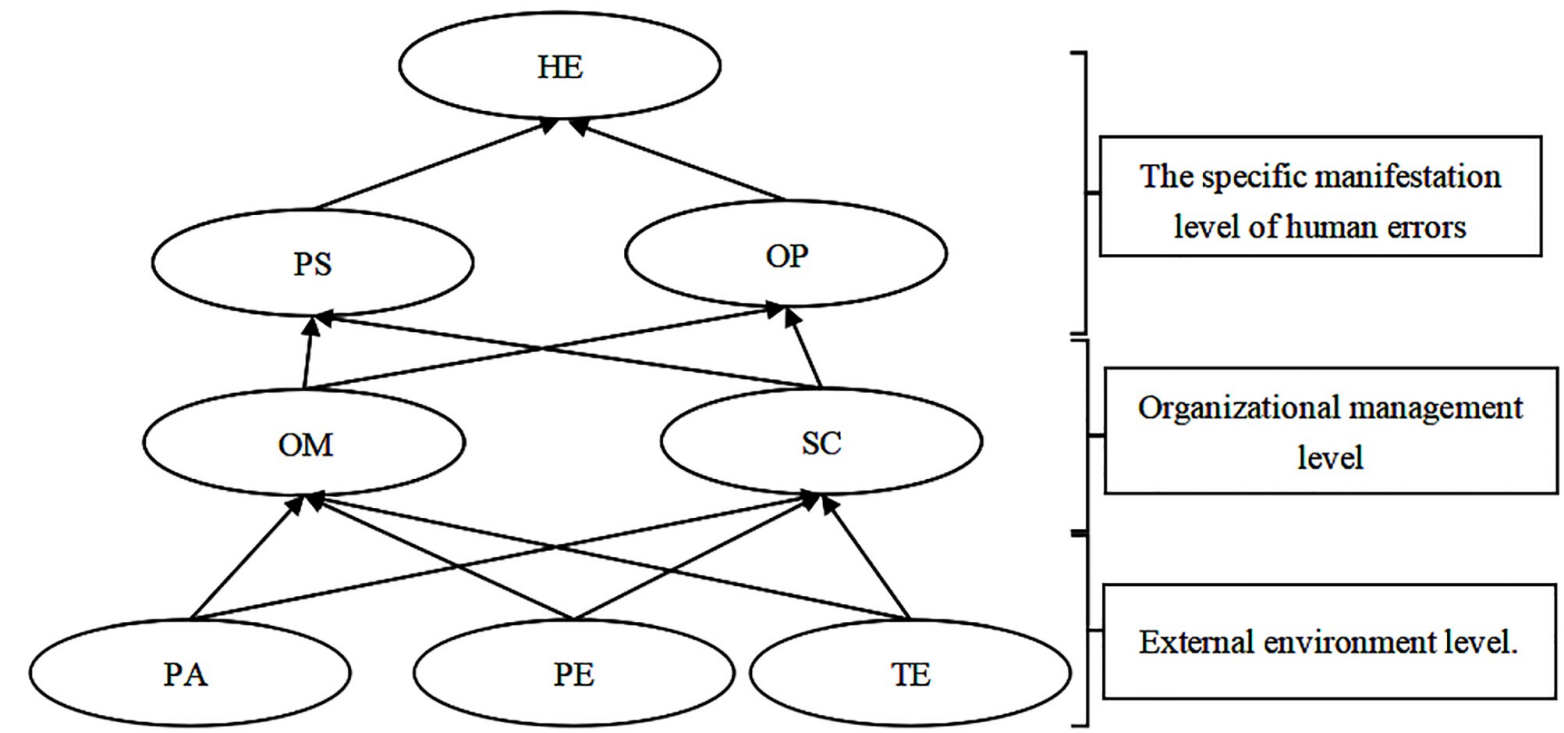
Multi-level hierarchical model of influencing factors for driver interface-related human errors. Note: HE is human error, PS is personal status, PA is personal ability, OM is organizational management, SC is safety culture, OP is operating procedures, PE is physical environment and TE is technical environment.

At the concrete manifestation level, personal state and operating procedures are specific factors that contribute to human errors. In the context of work, personal state can manifest as fatigue, loss of attention, and health issues caused by work-related factors. Operating procedures primarily involve driver non-compliance and failure to follow safety manuals, which directly lead to human errors. Non-compliance or improper adherence to personal state and operating procedures significantly impact the occurrence of human errors in tower crane operations, preventing effective accident prevention, control during operations, and post-incident mitigation. Therefore, efforts should be made to ensure the safety and stability of these factors. Addressing the hidden risks associated with the surface-level factors is crucial to safeguarding the drivers’ safety. Organizational management and safety culture are two key factors at the organizational management level. These factors play vital roles in the transmission of interactions between various influencing factors related to driver interface-related human errors in intelligent construction site tower crane operations. Thus, close attention and proper control of these factors are essential to positively influence the surface-level factors and gradually improve the issue of human errors in tower crane operations. Personal abilities, physical environment, and technological environment are external environmental factors. These factors possess characteristics of suddenness and wide-ranging effects, capable of significantly impacting tower crane operations. Therefore, preventive and contingency measures should be implemented to reduce the uncontrollability associated with these factors. The result is shown in Fig 4.

### Driver interface human error influencing factors BN model establishment and analysis

Bayesian Network (BN), also known as a belief network, consists of a Directed Acyclic Graph (DAG) and Conditional Probability Tables (CPT). The DAG describes the topological structure of the Bayesian network, including nodes representing variables and directed edges indicating causal relationships between nodes [53]. The topological structure obtained through Information Structure Matching (ISM) can be used to construct the Bayesian network, where ISM provides relationships between nodes that help determine the directed edges in the DAG [54]. In the DAG, if there is a directed edge from node x1 to node x2, then x1 is called the parent node of x2, and x2 is called the child node of x1. Nodes without parent nodes are referred to as root nodes, while nodes without child nodes are referred to as leaf nodes.CPT describes the conditional probabilities between nodes, expressing the strength of relationships between nodes. Each node in the CPT has corresponding conditional probabilities, calculated based on the Bayesian formula (Eq 10).


$$P(A|B)=\frac{P(A)P(B|A)}{P(B)}$$ (10)

In this formula, P(A) represents the prior probability, and P(A|B) represents the posterior probability. For root nodes, only the prior probabilities are calculated, while nodes with parent nodes require calculation of multivariate conditional probabilities. Based on the characteristics of the Bayesian network, when calculating the values of parent nodes, each node is independent of its parent nodes, and the multivariate conditional probabilities can be calculated using the law of total probability, as shown in Eq 11.


$$P(x_{1},x_{2},\ldots x_{n})=\prod_{i=1}^{n}P(x_{i}|Parent(x_{i}))$$ (11)

**Fig 5:**
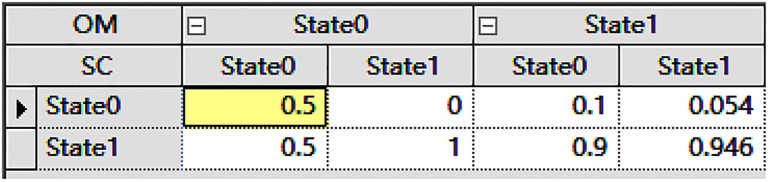
Partial conditional probability table.

There are two methods to obtain CPT tables: one is based on expert knowledge, but it has a subjective nature; the other is data-driven [55], with Maximum Likelihood Estimation (MLE) [56] being the most common in data-driven methods. This paper adopts the MLE method for data-driven approaches. The statistical analysis of human error behavior is conducted through relevant searches using terms such as "driver error", "driver behavior." and so on. CPT tables obtained through data-driven methods, which involve calculating conditional probabilities based on actual data, reflect the probability distribution relationships between nodes. Each entry in the CPT table represents the conditional probability of a specific node given the values of its parent nodes. By combining the topological structure provided by ISM and the data-driven CPT tables, a complete Bayesian network can be constructed for inferring relationships and probability distributions between nodes.As shown in Fig 5.

A Bayesian Network (BN) model was constructed based on the multi-level hierarchical structure of human error factors in intelligent construction site tower crane drivers, as shown in Fig 4. Before performing Bayesian network calculations, the word frequencies obtained from text mining were normalized using softmax to ensure that each frequency value falls between 0 and 1, and the sum of all frequency values equals 1. The purpose of normalization is to transform the original frequencies into probability distributions, representing the likelihood of various events or states. Normalizing the frequencies aligns them more closely with the definition of probability distributions and supports compatibility and consistency with Bayesian networks, providing a basis for subsequent inference, prediction, and analysis. Dimensionality Reduction Results of Normalized Intelligent Construction Site Tower Crane Accident Investigation Report Featuresare are shown in Table 9. Failure Probability of Driver Interface Human Error Influencing Factors’ Indicators are shown in Table 10.

**Table 9:** Dimensionality reduction results of normalized intelligent construction site tower crane accident investigation report features.

Impact Factors	Frequency	Impact Factors	Frequency	Impact Factors	Frequency
Feel unwell	0.0075	Teamwork	0.0073	Cockpit temperature	0.0037
Physical state	0.0136	Teamwork	0.0037	Cockpit humidity	0.0037
Illness	0.0037	Operating specification	0.0135	Illumination	0.0037
Health	0.0037	Operating system	0.0168	Hue	0.0037
Fatigued	0.0037	Rules and regulations	0.0037	Noise	0.0037
Dispersion of attention	0.0101	Reward and punishment system	0.0037	Vibration	0.0037
distract	0.0037	Management system	0.0037	Crossing condition	0.0074
Inattention	0.0037	Job training	0.0074	Construction site	0.0037
Emotional stability	0.0037	Safety education	0.0037	Site obstacle	0.0074
Testiness	0.0037	Job management	0.0037	Weather	0.0037
Safety awareness	0.0037	Operating procedure	0.0037	Digital interface display	0.0037
Professional skill	0.0038	Technical specification	0.0101	Digital interface information delivery	0.0037
Operational skill	0.0037	Regulation	0.0102	Information transmission	0.0101
Defense	0.0074	Long working hours	0.0037	Safety sign	0.0074
Safety belt	0.0037	The work schedule is not reasonable	0.0037	Display and control page layout	0.0037
Safety Helmet Hat	0.0037	Cable worker communication	0.0037	Display and control operation mode	0.0101
Safety measure	0.0037	Signalman	0.0037	Display and control device density	0.0037
Protective device	0.0037	Untimely signal	0.0037	Drive-by-wire reliability	0.0037
Working hours	0.0101	Improper command	0.0168	Space comfort	0.0037
Staffing	0.0076	Emergency drill	0.0067	Cockpit seat comfort	0.0037
Distribution of responsibilities	0.0074	Emergency plan	0.0037	Communication equipment	0.0037
Time pressure	0.0037	Preventive measures	0.0037	System intelligence	0.0075
Time shortage	0.0037	Working atmosphere	0.0102	System reliability	0.0037

**Table 10:** Failure probability of driver interface human error influencing factors’ indicators.

Index	Failure probability
Personal status	0.0571
Personal ability	0.0696
Organization management	0.0656
Safety culture	0.0522
Operating procedure	0.0654
Physical environment	0.0370
Technical environment	0.0758

Each influencing factor was set to have two states: occurrence (STATE(0)) and non-occurrence (STATE(1)). The normalized word frequencies obtained from text mining were summed according to the indicator layer of DEMATEL, and the obtained prior probabilities of parent nodes and conditional probability data between nodes were imported to generate the Conditional Probability Tables (CPT) that reflect the dependencies between response nodes. According to statistics, the number of accidents involving tower cranes in China has been decreasing in recent years. The majority of accidents are primarily caused by a lack of personal safety awareness and violations of regulations and rules, making human error the main cause of these accidents [4].

## Case calculation

### Incident background

An accident involving a tower crane occurred at a smart construction site in Guangxi. It was reported that the tower cranes at the site were equipped with hook visualization. On December 19, 2022, at 8:00 AM, the carpentry team leader and eight employees arrived at the construction site in Guiping City Chang’an Industrial Park Yuanantang Project to assemble scaffolding.

At around 8:30 AM, the tower crane operator climbed up the tower crane to inspect the repair status as it had undergone maintenance on December 18. After about ten minutes, the construction supervisor and safety officer from a subcontracting company in Nanning contacted the tower crane operator via intercom, instructing them to use the tower crane to unload the steel pipes from the trailer onto the open space near the base of the tower crane, with a small portion being unloaded at the scaffolding location.

At around 9:40 AM, the safety officer asked the carpentry team leader to arrange personnel to assist in unloading the steel pipes from the trailer. The team leader assigned a scaffolder to help, who used the tower crane’s lifting rope to secure and bind the steel pipes (about 30 pounds each, approximately 5.2 meters long, with a diameter of approximately 50 millimeters) bundled with steel wire ropes and U-shaped clips (40–50 pipes per bundle). The temporary signal worker in charge directed the tower crane operator to lift the pipes. At that time, there were more than ten workers constructing the scaffolding in the swinging range of the tower crane.

At around 11:00 AM, after multiple lifts, the tower crane lifted the steel pipes to a height of about 20 meters and moved the jib horizontally by about 10 meters. At that moment, the U-shaped clips securing the steel wire ropes of the bundled pipes came loose (during this lift operation, the pipes were not suspended using the tower crane’s lifting ropes directly but rather the ropes were threaded through the steel wire ropes of the bundled pipes), causing the pipes to fall and strike Employee A, who was working below the jib, on the head, resulting in severe injuries and loss of consciousness. The scattered reinforcing bars also struck Employee B’s foot. The on-site personnel immediately called emergency services (120 and 110) and reported the incident to the project manager. After more than ten minutes, medical personnel from the emergency services arrived, followed by the police, who initiated rescue and treatment. After more than 20 minutes of treatment, the on-site medical personnel declared the death of Liao Jianjian and transported the lightly injured Employee B to the People’s Hospital in Guiping City for further medical care.

## Analysis and calculation

Taking the tower crane accident at the smart construction site as the evaluation object, the normalized text mining frequencies are integrated with a Bayesian Network (BN) to calculate the probability of this incident occurring.

**Fig 6:**
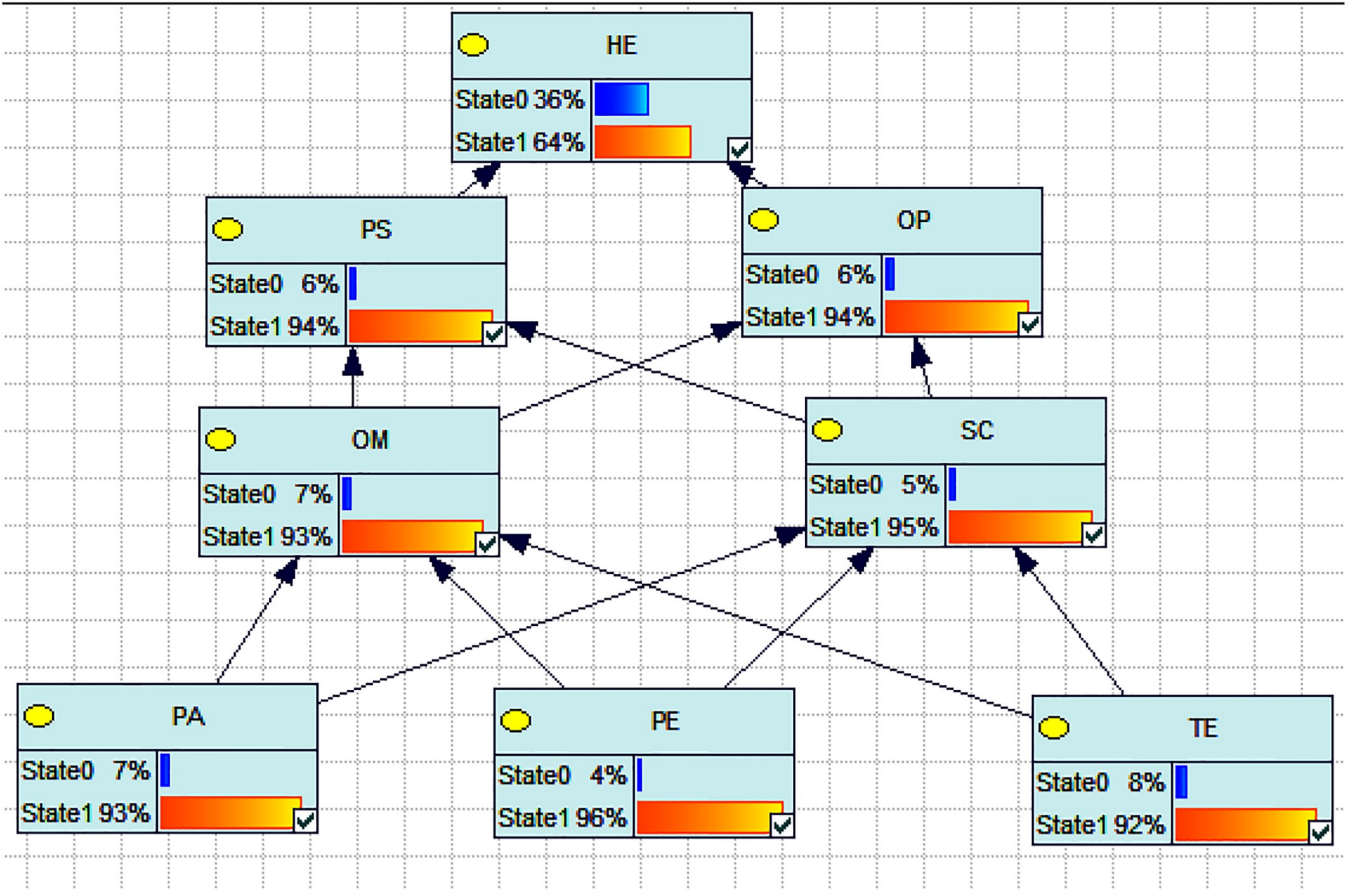
Human errors causes causal reasoning. Note: HE is human error, PS is personal status, PA is personal ability, OM is organizational management, SC is safety culture, OP is operating procedures, PE is physical environment and TE is technical environment.

Based on the incident background, the reliability probability of the human-machine interface factor in the driver’s cabin was calculated as 36% using GeNIe2.0 software, as shown in Fig 6. This validates the applicability of the proposed DEMATEL-ISM-based Bayesian Network method for evaluating the reliability of the human-machine interface factor in tower crane drivers at smart construction sites. According to the figure, the highest probability of failure among the human-machine interface factors in tower crane drivers at the smart construction site is the occurrence of technological environment failure, which is 8%. This is followed by the probability of personal ability failure at 7%,organizational management failure at7%,personal status, operating procedures failure at 6%, and safety culture failure at 5% and physical environment failure at 4%.

**Fig 7:**
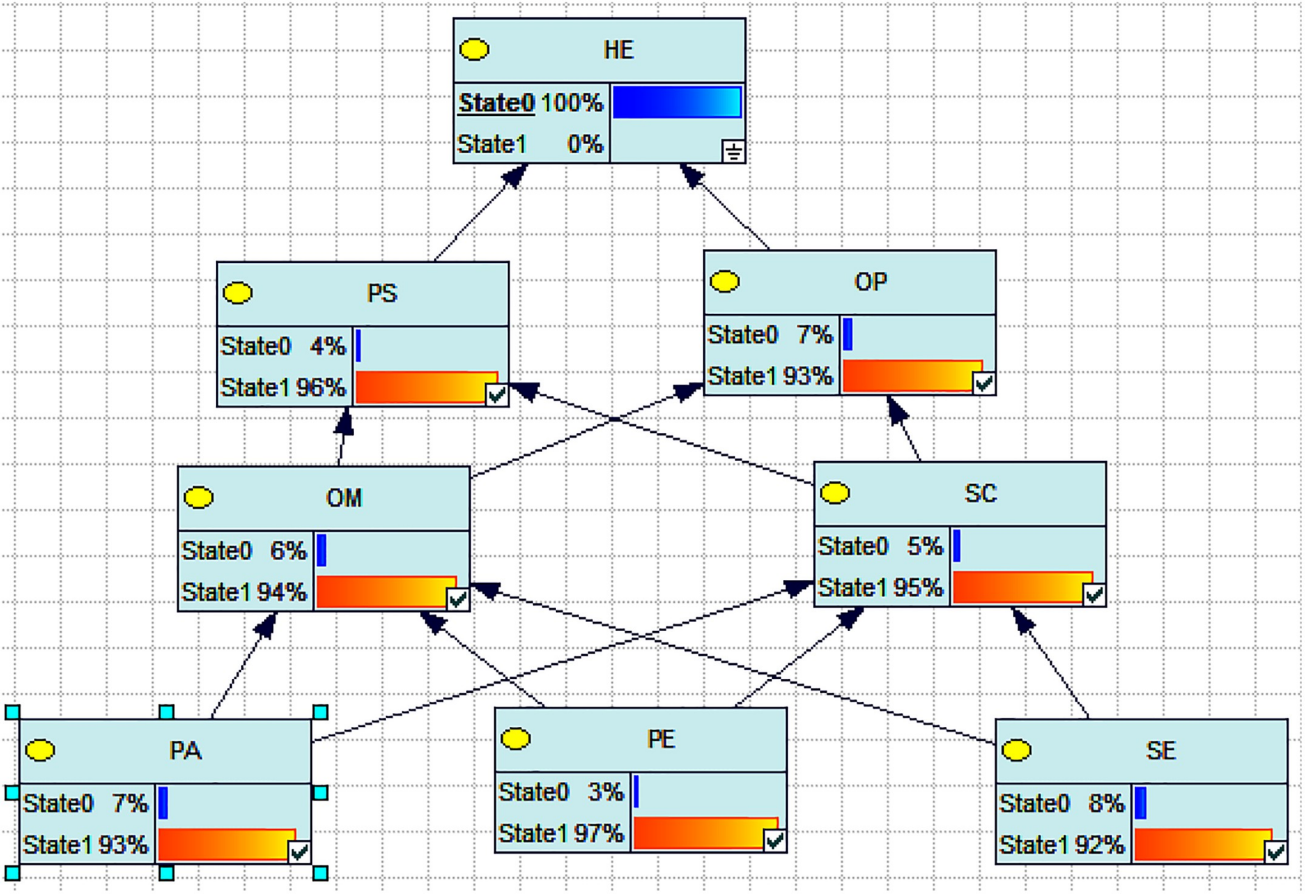
Probability distribution of other nodes when human error occurs. Note: HE is human error, PS is personal status, PA is personal ability, OM is organizational management, SC is safety culture, OP is operating procedures, PE is physical environment and TE is technical environment.

The Bayesian network model built using GeNIe software can analyze the probabilities of factors contributing to human interface errors when they occur. Through reverse inference, the model can determine the main causal pathway leading to the occurrence of human interface errors in tower crane operator accidents based on the probabilities of network nodes. The main process is as follows: setting human interface errors as a certain state, i.e., "State0 = 100%", updating the network model, and determining the probabilities of related nodes occurring under this condition. Starting from the factor node, the pathway of the main causal factors leading to the occurrence of the factor is determined layer by layer based on the causal relationship. As shown in Fig 7,the following pathway: Technical Environment → Organizational Management → Operating Procedures → Human Interface Errors. The technical environment consists of the hardware equipment in the driver’s cabin, and a superior technical environment can provide tools, resources, data processing capabilities, and communication mechanisms while supporting automation, standardization, safety, and risk management. These factors collectively influence the formation of an company’s organizational management. Companies need to consider and utilize the characteristics of the technical environment as key elements for management and the development of safety culture. The technical environment in tower crane operations is the foundation of driver’s operating procedures, and it significantly influences the operating procedures. The following aspects of the technical environment affect the operating procedures: automation level, visualization and interface design, data collection and monitoring, human-machine interaction, fault handling and emergency management, and safety measures. The technical environment has a significant impact on the content, processes, user-friendliness, and safety of operating procedures. When developing operating procedures, the current technical environment needs to be considered, and the procedures should be adjusted accordingly to leverage and adapt to the characteristics and functionalities of the technical environment. Therefore, the pathway indicates that the quality of the technical environment can directly impact the companies’ organizational management, which in turn affects the occurrence of human interface errors in accordance with driver’s operating procedures, leading to tower crane accidents.

In order to identify the factors that have the greatest impact on the probability of human interface errors, in the Bayesian network model, each influencing factor is successively set to "State1 = 100%", indicating the non-occurrence or control of the influencing factor. Through simulation using GeNIe software, the probabilities of human interface errors occurring under the non-occurrence of each influencing factor are determined, as shown in Table 11. From the table, it can be seen that improving personal state and technical environment can significantly reduce the probability of interface-related human errors.

**Table 11:** Probabilities of human interface errors after simulation.

IF	HRA (%)
PS	37
PA	36
OM	36
SC	36
OP	36
PE	36
TE	36

Note: HE is human error, PS is personal status, PA is personal ability, OM is organizational management, SC is safety culture, OP is operating procedures, PE is physical environment and TE is technical environment

Sensitivity values can measure the impact of changes in the upper-level nodes on the probability of the target node in a network structure. The higher the sensitivity value, the more pronounced the effect. The sensitivity values of the upper-level nodes are obtained through sensitivity analysis, and a larger sensitivity value indicates that the node is a critical node. In GeNIe software, after conducting sensitivity analysis, nodes that tend towards dark red color indicate higher values, while black and gray nodes represent a sensitivity value of 0.

**Fig 8:**
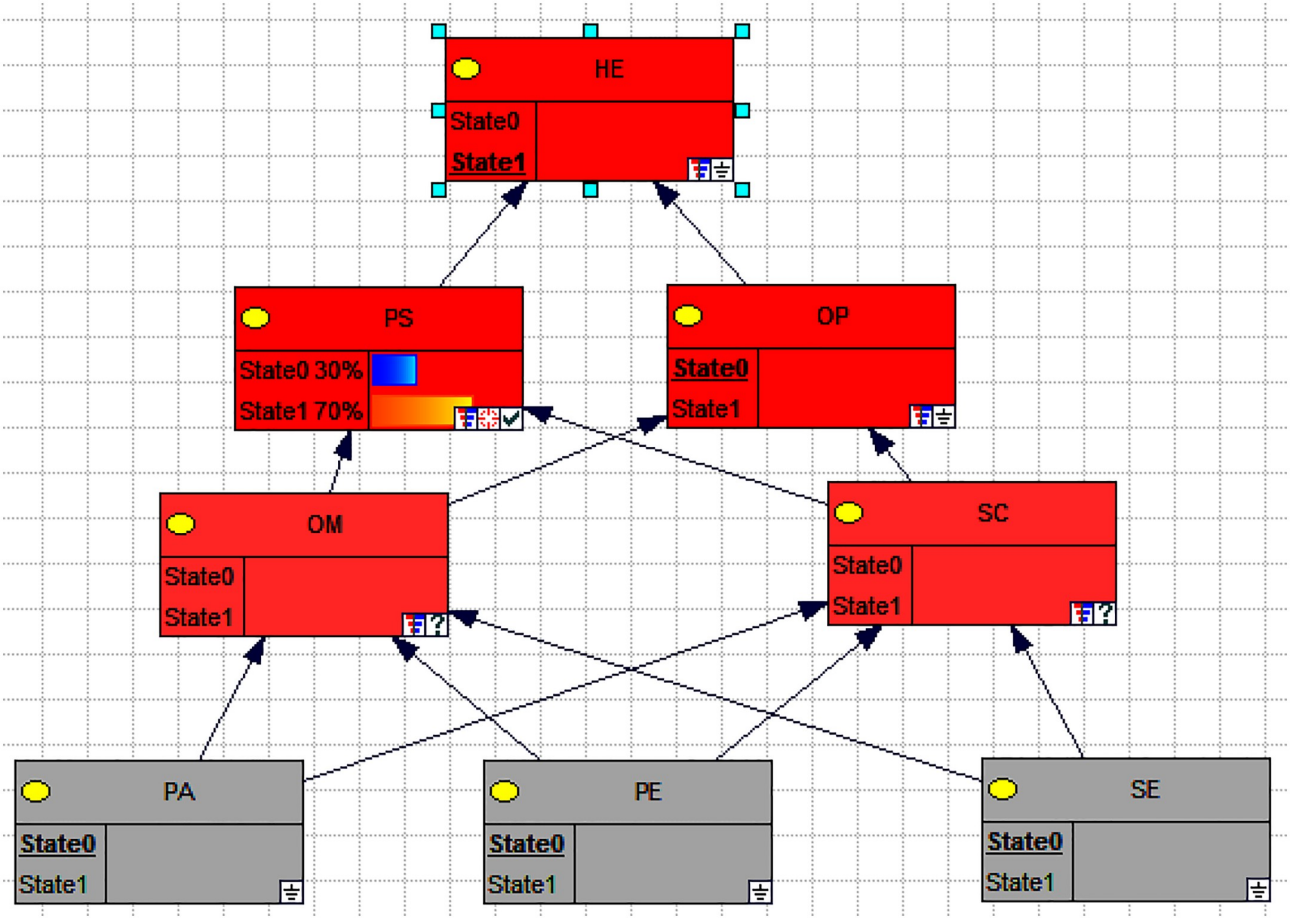
Human error sensitivity analysis diagram. Note: HE is human error, PS is personal status, PA is personal ability, OM is organizational management, SC is safety culture, OP is operating procedures, PE is physical environment and TE is technical environment.

For example, taking the target node as "HE" (possibly referring to Human Error), the sensitivity analysis results shown in the Fig 8 indicate that personal status and operating procedures are represented in deep red color, indicating that these two factors have the most direct influence on human errors. Organizational management and safety culture are represented in pink color, indicating that although these two factors do not have a direct impact, they have a profound influence as they directly affect personal status and operating procedures.

## Conclusion and recommendations

The study focused on the frequent occurrence of human-induced interface errors among drivers of smart construction site tower cranes. Various methods were employed, including text mining, the SHEL model, the DEMATEL model, structural equation modeling, and Bayesian networks. By exploring the causal factors of accidents in tower crane case reports, a list of factors influencing human error in smart construction site tower crane accidents was obtained. Based on the SHEL model, the factors influencing human error were categorized hierarchically. The DEMATEL-ISM model was then used to conduct a systematic analysis of the factors influencing human-induced interface errors in tower crane drivers. Quantitative analysis was performed to reveal the complex hierarchy and causal paths among the factors. The results showed that human error, personal state, and operating procedures constituted the performance layer of human-induced errors, while safety culture and organizational management belonged to the organizational management layer, and personal capability, physical environment, and technological environment constituted the external environment layer. Subsequently, the multi-level hierarchical structure model obtained from DEMATEL-ISM was used as a prototype for establishing a Bayesian network. The previously mined word frequency from text mining was normalized using Softmax, and the normalized failure probability was combined with the Bayesian network. Finally, through the analysis and verification of a specific crane accident case, the feasibility of this method in calculating human reliability for smart construction site tower cranes was validated. The main conclusions are as follows:

1. Firstly, text mining (TF-IDF-TruncatedSVD-ComplementNB) was used to analyze the causes of tower crane accidents from 2018 to 2023, clearly identifying human error as the main factor leading to interface accidents in tower cranes. In this study, focusing on smart construction site tower cranes, a text mining approach (using TF-IDF-TruncatedSVD-ComplementNB) was employed to successfully identify the patterns of human error behavior in tower crane drivers based on the analysis of 229 accident reports from traditional tower cranes and smart tower cranes.
2. Combining the SHEL method with factors obtained through text mining, we can correlate the influencing factors with the SHEL behavior factors to ensure a more objective selection of influencing factors. Relevant failure modes and potential causes can be found in the literature to support this process. A questionnaire based on the forming factors is then sent to 137 professional tower crane operators. Through reliability and validity tests, items with scores lower than 0.5 are eliminated.
3. Considering the complexity of comparing 27 forming factors with each other, it would be cumbersome to create 676 items in the expert questionnaire, and this could lead to score distortion. Therefore, based on expert experience and literature research, the 27 forming factors are consolidated into 7 constructs. The DEMATEL-ISM model is employed for a systematic analysis of the influencing factors of human errors in the intelligent construction site tower crane driver interface. The quantitative analysis reveals the complex hierarchical structure and causal relationships among the factors. The reachable matrix is partitioned into levels using the Spectral Clustering algorithm and expert experience. The results indicate that human errors, personal states, and operating procedures represent the performance layer of human errors, while safety culture and organizational management represent the organizational management layer, and personal abilities, physical environment, and technological environment represent the external environmental layer.
4. Subsequently, the hierarchical structure model obtained from DEMATEL-ISM is used as a basis for constructing a Bayesian network. To ensure objectivity, rigor, and applicability of the data for the Bayesian network, the word frequencies obtained from previous text mining are normalized using Softmax, and the normalized failure probabilities are combined with the Bayesian network. Finally, through analysis and validation of a specific crane accident case, the feasibility of this method in calculating human reliability in the application of smart construction site tower cranes is verified.
5. From the multi-level hierarchical structure obtained from DEMATEL-ISM-BN, it can be observed that human errors occur as a result of multiple factors interacting and coupling with each other. Personal states and operating procedures are direct influencing factors of human errors. Personal states and operating procedures are directly influenced by safety culture and organizational management, while technological environment, physical environment, and personal abilities directly influence safety culture and organizational management. This indicates that each factor contributing to human errors is interconnected, and if any indicator is inadequate, it may lead to a butterfly effect, triggering more serious accidents. Furthermore, the technological environment, as a vulnerable point requiring frequent updates, requires increased observation and investment from companies to prevent human errors from occurring at the most basic level.

Through analysis and calculations of accident cases, it can be concluded that the company has inadequate safety education and training for employees, resulting in a lack of knowledge regarding safety production. This hinders their ability to identify accident hazards or other unsafe factors, leading to insufficient accident prevention capabilities. Additionally, there are instances of improper command and risky operational practices. This results in inadequate management of construction sites, as the company fails to timely discover and eliminate accident hazards present at the work site, and does not take action to stop or rectify unsafe behaviors such as employees giving improper instructions or engaging in risky operations.

## Supporting information

S1 File (RAR)
